# Cross-reactivity of antibodies to different rumen methanogens demonstrated using immunomagnetic capture technology

**DOI:** 10.3389/fmicb.2022.918111

**Published:** 2022-08-22

**Authors:** Sofia Khanum, Joanna M. Roberts, Rosemary W. Heathcott, Stefanie Bagley, Tania Wilson, Sandeep K. Gupta, Michelle R. Kirk, Axel Heiser, Peter H. Janssen, D. Neil Wedlock

**Affiliations:** ^1^AgResearch Ltd., Palmerston North, New Zealand; ^2^Flowjoanna, Palmerston North, New Zealand

**Keywords:** magnetic beads, methanogens, rumen, immunomagnetic capture technology, antibodies

## Abstract

Methane is produced in the rumen of ruminant livestock by methanogens, accounting for approximately 14.5% of anthropogenic greenhouse gas emissions in terms of global warming potential. The rumen contains a diversity of methanogens species, and only a few of these have been cultured. Immunomagnetic capture technology (ICT) is a simple and effective method to capture and concentrate target organisms in samples containing complex microflora. We hypothesized that antibody-coated magnetic beads could be used to demonstrate antibody specificity and cross-reactivity to methanogens in rumen samples. Sheep polyclonal antibodies raised against four isolates of rumen dwelling methanogens, *Methanobrevibacter ruminantium* strain M1, *Methanobrevibacter* sp. AbM4, *Methanobrevibacter* sp. D5, and *Methanobrevibacter* sp. SM9 or an equal mix of all four isolates, were used to coat paramagnetic beads. ICT was used together with flow cytometry and qPCR to optimize key parameters: the ratio of antibody to beads, coupling time between antibody and paramagnetic beads to produce immunomagnetic beads (IMBs), and optimal incubation time for the capture of methanogen cells by IMBs. Under optimized conditions, IMBs bound strongly to their respective isolates and showed a degree of cross-reactivity with isolates of other *Methanobrevibacter* spp. in buffer and in rumen fluid, and with resident methanogens in rumen content samples. The evidence provided here indicates that this method can be used to study the interaction of antibodies with antigens of rumen methanogens, to understand antigen cross-reactivity and antibody binding efficiency for the evaluation of antigens used for the development of a broad-spectrum anti-methanogen vaccine for the abatement of methane production.

## Introduction

Methane produced in the rumen of ruminant livestock accounts for approximately 14.5% of global anthropogenic greenhouse gas emissions and 40% of all livestock emissions (Gerber et al., 2013), and represents a loss of 2-12% of ingested energy in ruminants (Johnson and Johnson, 1995). Hydrogen and methyl compounds are produced during fermentation of ingested feed by a subset of the fermentative community of bacteria, protozoa and fungi in the rumen. These intermediates are used by methanogenic archaea (methanogens) as energy sources and converted to methane (Janssen, 2010). Methane cannot be used by the ruminant and is removed from the rumen by eructation into the atmosphere. The diversity of methanogens in the rumen is relatively limited in comparison to bacteria and affected by inter-animal variation, diet, geographical region and rumen sampling (Wright et al., 2007; Jeyanathan et al., 2011; Henderson et al., 2015). The great majority of ruminal methanogens belong to two orders: *Methanobacteriales* and *Methanomassiliicoccales* (Henderson et al., 2015). The genus *Methanobrevibacter*, a member of the family *Methanobacteriaceae* in the *Methanobacteriales*, appears to dominate the rumen globally, making up about 76% of rumen archaea (Henderson et al., 2015). It is unclear how many species there are, but many are not available as laboratory cultures (Seedorf et al., 2014). Pure-culture isolates have been assigned to a number of named species, including *M. ruminantium*, *M. olleyae*, *M. gottschalkii*, *M. thauerii*, *M. millerae*, *M. boviskoreani*, *M. wolinii*, and *M. smithii*. Another genus in the *Methanobacteriaceae* is *Methanosphaera*, which may make up some 8% of rumen methanogens (Seedorf et al., 2014; Henderson et al., 2015).

Phenotypically, the abundant rumen methanogens fall into two broad groups: hydrogenotrophic methanogens and hydrogen-requiring methylotrophic methanogens. Hydrogenotrophic methanogens convert hydrogen and sometimes formate to methane, and this is the physiology of *Methanobrevibacter*. The hydrogen-requiring methylotrophic methanogens belong to the genus *Methanosphaera* and the diverse assemblage of rumen methanogens in the family *Methanomassiliicoccaceae* within the order *Methanomassiliicoccales* (Borrel et al., 2014). The hydrogenotrophic methanogens are estimated to be dominant in terms of both methane emissions and community composition (Henderson et al., 2015).

Multiple strategies are under investigation to mitigate ruminant methane production. Some of these have focused on direct inhibition of methanogens using chemical inhibitors, while others are based on feed or natural products in the feed components (Hristov et al., 2015; Sun et al., 2015; Beauchemin et al., 2020). In previous studies, administration of chemical inhibitors such as bromochloromethane and 3-nitrooxypropanol has reduced methane emissions by directly inhibiting methanogen growth in the rumen (Abecia et al., 2012; Hristov et al., 2015). Breeding for low-emitting ruminants is another promising approach, which fits well with farming practices without having to change farm systems (Pickering et al., 2015). Another attractive approach that does not require a change to farm management is to develop a broad-spectrum anti-methanogen vaccine to reduce methane emissions from ruminants (Wedlock et al., 2013).

A vaccine has the potential to harness an immune response to limit the growth of specific targeted rumen methanogens without negatively impacting animal health (Wedlock et al., 2010). The aim is to generate antibodies that are delivered into the rumen via saliva, where they interact with and impair the metabolism of methanogens and thereby reduce their ability to form methane. Vaccination of sheep with *M. ruminantium* resulted in the induction of specific antibodies in serum and saliva. Serum antigen specific antibodies reduced the growth of these microbes in an *in vitro* assay (Wedlock et al., 2010). Antibodies enter the rumen via saliva and can be detected in rumen content samples from the vaccinated animals (Wedlock et al., 2013; Subharat et al., 2015; Subharat et al., 2016). Previously, animals vaccinated against the rumen-dwelling bacterium *Streptococcus bovis* were shown to control its deleterious effects on animals fed grain-rich diets (Gill et al., 2000), demonstrating the potential for this approach.

To develop a broad-spectrum effective vaccine, it is important to consider the phylogenetic and physiological diversity over the different orders of methanogens and include vaccine antigens that are cross-reactive for a wide range of rumen dwelling methanogens. We sought a method to evaluate the specificity of antibodies generated against methanogens and also to concentrate the targeted methanogens from a pool of a wide range of methanogens resident in the rumen. We therefore used immunocapture technology (ICT), which employs antibody-coated paramagnetic beads, to identify their specificity for methanogens in complex rumen samples. ICT is a simple and effective method of selectively capturing and concentrating microbes from complex microbial communities (Varshney et al., 2005; Wang et al., 2011). Immunomagnetic beads (IMBs) are magnetic particles coated with antibodies against target organisms. The specificity of the antibodies coupled with the magnetic properties of the beads allows target organisms to be easily separated and concentrated from complex microbial communities and background microflora (Diler et al., 2011). In future, this method could be used to understand antigen cross-reactivity, antibody binding efficiency toward cultured and uncultured methanogens in rumen contents and for evaluating antigens for use in a broad-spectrum vaccine for reducing methane production.

## Materials and methods

### Methanogen isolates and cultural conditions

Methanogen isolates used in this study are listed in Table 1. Each isolate was stored at −80°C in the AgResearch collection prior to use. Isolates were grown from frozen stocks anaerobically in BY medium in 10-ml volumes in Hungate tubes or 60-ml volumes in 125-ml serum vials under a CO_2_ headspace (Joblin, 2005), to which H_2_ gas was added to 1.4 bar to facilitate methanogen growth. Cells in late-logarithmic to early stationary phase cultures were harvested at OD_600_ 0.4 by centrifugation at 13,000 *g* for 20 min. Cell pellets were washed three times with phosphate-buffered saline (PBS, 10 mM, pH 7.3) prior to formulating whole cell vaccines for producing antisera in sheep and for *in vitro* methanogen capturing experiments.

**TABLE 1:** Methanogen isolates.

Isolate	Description	Reference and source
*M. ruminantium* M1	Bovine rumen isolate	Smith and Hungate, 1958, DSMZ*^a^*
*Methanobrevibacter* sp. AbM4	New Zealand sheep abomasum isolate	Leahy et al., 2013, AgResearch collection
*Methanobrevibacter* sp. D5	New Zealand sheep rumen isolate	Li, 2016, AgResearch collection
*Methanobrevibacter* sp. SM9	New Zealand sheep rumen isolate	Kelly et al., 2016, AgResearch collection

^a^DSMZ, Deutsche Sammlung von Mikroorganismen und Zellkulturen, Braunschweig, Germany.

### Production of antisera in sheep and collection of sera and rumen contents

Animal ethics approval for the trial was granted by the Grasslands Animal Ethics Committee (Palmerston North, New Zealand). Antisera were produced against each of the four methanogen isolates. Groups (*n* = 3) of Romney sheep (6 months of age, grazing ryegrass dominated pasture) were vaccinated twice by the intramuscular route at a 4-week interval with whole cell preparations of either *M. ruminantium* M1 (DSM 1093; (Smith and Hungate, 1958), *Methanobrevibacter* sp. AbM4 (Leahy et al., 2013), *Methanobrevibacter* sp. D5 (Li, 2016), *Methanobrevibacter* sp. SM9 (Kelly et al., 2016) or with an equal mix of all four isolates (MIX4). Each dose of the vaccine consisted of approximately 10^9^ cells (5 mg of wet weight cell pellet). The whole cell antigens were formulated with Montanide ISA61 adjuvant (SEPPIC, Paris, France) to enhance immunogenicity. A control group (*n* = 3) received a vaccine containing only adjuvant.

Sera were collected in serum-separation tubes (BD Vacutainer; BD Biosciences, San Jose, CA, United States) prior to vaccination and at 2 weeks after the second vaccination (week 6). Antibody titers in sera were measured by ELISA as described previously (Wedlock et al., 2010). Prior to coating beads, the IgG in each serum sample was enriched using a HiTrap protein G HP column (GE HealthCare, Danderyd, Sweden) according to the manufacturer’s protocol.

Rumen content samples were collected from the control sheep vaccinated with Montanide adjuvant alone by insertion of a stomach tube without a terminal strainer and application of suction within 2-3 h after they had last consumed grass. The rumen content samples were filtered through a single layer of cotton cheesecloth with a mesh size of approximately 1 mm to remove plant material and kept on ice until brought back to the laboratory. Sterile rumen fluid was obtained by centrifugation of the rumen content samples at 4,000 g for 20 min and filtering the supernatant through a syringe filter with a nominal pore size of 0.2 μm (Corning, Hamburg, Germany). Fresh rumen content and sterile rumen fluid samples were used for all experiments.

### Preparation of immunomagnetic beads

Tosylactivated paramagnetic beads (Dynabeads^®^ MyOne™ Tosylactivated, 1 μm diameter) were obtained from Invitrogen (Life Technologies, Auckland, New Zealand) and coated with enriched IgG according to the manufacturer’s protocol. Briefly, beads (1 × 10^12^ beads/ml) were resuspended by vortexing for 1 min. From this stock, 50 μl (containing 5 × 10^9^ beads) was transferred to a 2 mL Eppendorf tube and combined with 1 mL coating buffer (0.1 M sodium borate, pH 9.5). Beads were concentrated in a magnetic separator rack (DynaMag-2; Thermofisher Scientific, Auckland, New Zealand) for 2 min. After gentle removal of the supernatant, pellets were resuspended in coating buffer containing ligand antibody (total IgG-antibodies generated by vaccination) at a concentration of ∼40 μg antibody/mg beads. A 3 M ammonium sulfate stock solution, prepared in 0.1 M sodium borate buffer, pH 9.5, was added to the bead-antibody mix according to the manufacturer’s protocol and the bead suspensions were incubated for 16 h at 37°C with slight tilting rotation. Following incubation, supernatants containing unbound antibodies were removed by placing the tubes on the magnetic separator rack to collect and separate the beads from the suspensions. Supernatants were used to determine the concentration of unbound antibodies using a Pierce BCA protein assay kit (Thermofisher Scientific). To decrease non-specific binding reactions, beads were then resuspended in the same volume of blocking buffer (PBS containing 0.5% [w/v] BSA and 0.05% [v/v] Tween 20) and incubated at 37°C for 16 h with slight rotation, then washed three times with washing/storage buffer (PBS containing 0.1% [w/v] BSA and 0.05% [v/v] Tween 20). Following the washing step, beads were resuspended at a concentration of 5 × 10^6^ beads/μL in 1 mL storage/washing buffer and stored at 4°C until used. Beads prepared in this manner were designated immunomagnetic beads (IMBs).

### Confirmation of conjugation of antibodies to immunomagnetic beads

Binding of antibodies to paramagnetic beads was confirmed using M1-IMBs (IMBs coated with an IgG-enriched fraction from antisera produced against *M. ruminantium* M1). M1-IMBs (1 × 10^8^/ml) were mixed with either a 1:100 dilution of fluorescein isothiocyanate (FITC)-labeled anti-sheep antibody (donkey anti-sheep IgG (H + L)-FITC, Thermofisher Scientific), or FITC-labeled anti-mouse antibody (goat anti-mouse IgG F(ab’)2-FITC, Thermofisher Scientific) in a 100 μL volume and incubated at room temperature for 1 h with mixing by continuous rotation. The IMBs were recovered using the magnetic separator rack, washed three times with 1 mL storage/washing buffer, resuspended in 100 μL PBS, and used for flow cytometry analysis.

### Immunomagnetic bead capture of methanogens

Immunomagnetic capturing technology (ICT) was carried out on pure methanogen cell cultures resuspended in PBS and in freshly obtained rumen fluid and rumen content samples. PBS-washed cell suspensions of methanogens (1 × 10^7^ cells; estimated from OD_600_) were added to 1 × 10^8^ IMBs in a final reaction volume of 500 μL consisting of either PBS or rumen samples and incubated at room temperature for 1 h with continuous rotation. After incubation, the IMBs were recovered from the mixture using the magnetic rack described above, and then washed three times with 1 mL storage/washing buffer containing 0.5 M NaSCN, and finally resuspended in 200 μL PBS, and used for genomic DNA isolation and qPCR analysis.

### Genomic deoxyribonucleic acid isolation

Total DNA was extracted from the captured methanogens as described previously (Kittelmann and Janssen, 2011). Briefly, cells bound to IMBs were disrupted by combining bead-beating (FastPrep FP120; Qbiogene, Carlsbad, CA, United States; 45 s at 6.5 ms^–1^) and phenol-chloroform-isoamyl alcohol (25:24:1; v:v:v) treatment. Proteins were precipitated using chloroform-isoamyl alcohol (25:1; v:v) followed by centrifugation for 20 min at 14,000 g at 4°C. DNA was extracted from the supernatants using a QIAquick PCR purification kit (QIAGEN, Germantown, MD, United States) and stored at −20°C for qPCR.

### qPCR

Methanogens captured by IMBs were quantified using a Rotor-Gene 6000 real-time rotary analyzer (Corbett Life Science, Concorde, NSW, Australia) with amplicon detection using SYBR Green I fluorescence (Light Cycler Fast-Start DNA Master SYBR Green I Kit; Roche, Auckland, New Zealand). The extracted DNA was used as a template for PCR to amplify the archaeal 16S rRNA gene using forward primer Ar915af targeting 16S rRNA gene nucleotide positions 915 – 934 (5′-AGGAATTGGCGGGGGAGCAC-3′; Casamayor et al., 2002) and reverse primer Ar1386R targeting positions 915-934 (5′- GCGGTGTGTGCAAGGAGC-3′; Skillman et al., 2004). Nucleotide positions are based on the reference published in Lane (1991). Plasmids containing archaeal 16S rRNA gene inserts were constructed, quantified with the Quant-iT dsDNA BR Assay Kit on a Qubit fluorometer (Invitrogen, Carlsbad, CA, United States), and diluted 10-fold in series to produce five standards ranging from 2 × 10^7^ to 2 × 10^3^ copies per reaction, each in duplicate for use in the qPCR. Reactions were set up in PCR tubes. Each reaction of 20 μL contained 10 μL Light Cycler Mix, 5 μM of each primer, and 2 μL of standard or DNA template. The thermal protocol for qPCR amplification and detection was 10 min of initial denaturation (95°C), followed by 40 amplification cycles [10 s at 95°C; 5 s at 59°C; 10 s at 72°C]. After each run, melting curves between 72°C and 95°C were evaluated to confirm the absence of non-specific signals.

### Fluorescence microscopy

Fluorescence microscopy was used to visualize *M. ruminantium* M1 cells bound to IMBs by detecting the presence of intrinsic fluorescent cofactor F_420_ in methanogenic archaea (Cheeseman et al., 1972). F_420_ autofluorescence was visualized using a Leica DM2500 fluorescence microscope (Leica, Wetzlar, Germany) with a UV filter (excitation BP 355-425 nm).

### Flow cytometry

To detect coupling to IMB of antibodies purified from sheep sera, secondary fluorescein isothiocyanate (FITC) labeled anti-sheep antibodies (Abcam, Cambridge United Kingdom) were used. A FITC-labeled anti-mouse antibody (Sigma-Aldrich, St Louis, MO, United States) was used as a negative control. To detect attachment of M1 cells to antibody coated IMB, M1 cells were identified by using the 405nm laser to excite of the auto fluorescent methanogen coenzyme F_420_ and fluorescence was collected through a 448/45 band pass (BP) filter. IMBs were identified based on autofluorescence excited by the 488 nm laser and measured through a 750/54 BP filter. M1 cells attached to IMBs produced a signal in both detectors. Data were acquired and analyzed using a BD FACSVerse (BD Biosciences, San Jose, CA, United States) and FlowJo™ Software for Windows, Version (FlowJo_V10) (Becton, Dickinson and Company, Ashland, OR, United States).

### Cross-reactivity of sheep antibodies produced against individual methanogen isolates

The cross-reactivity of the antibodies produced against individual methanogens (*M. ruminantium* M1, *Methanobrevibacter* sp. AbM4, *Methanobrevibacter* sp. D5, and *Methanobrevibacter* sp. SM9) was determined using IMBs. A total of 1 × 10^8^ IMBs coated with antibodies produced against the four individual methanogen isolates or an equal mixture of antibodies against the four isolates (MIX4) were incubated with 1 × 10^7^ methanogen cells for 1 h at room temperature with slight rotation. Captured methanogens were washed and subjected to genomic DNA isolation for quantification by qPCR as described above. The identities of the methanogens captured with MIX4 IMBs were determined by 16S rRNA gene sequencing. The 16S rRNA reads were converted to cells based on the number of 16S rRNA genes per genome: *M. ruminantium* M1 and *Methanobrevibacter* spp. SM9 and D5 each have 2 (Leahy et al., 2010; Kelly et al., 2016; Li, 2016) while *Methanobrevibacter* sp. AbM4 has 3 (Leahy et al., 2013).

### Capture of methanogens in rumen content samples

Rumen content samples were obtained and prepared as described in section 2.2. Samples were diluted 1:1 with PBS containing protease inhibitors (Complete Inhibitor; Roche Diagnostics, Auckland, New Zealand) and incubated at room temperature for 1 h. A total of 1 × 10^8^ IMBs were added to 1 mL of protease inhibitor treated rumen content sample and the mixture was incubated for 1 h at room temperature with slight rotation. Captured methanogens were washed as described above followed by genomic DNA isolation, prior to quantification with qPCR, and identification by 16S rRNA gene sequencing.

### 16S rRNA sequencing and data analysis

Archaeal 16S rRNA genes were amplified by PCR from genomic DNA extracted from captured methanogens, as described previously (Kittelmann et al., 2015). The primers used were 5′-CAA GCA GAA GAC GGC ATA CGA GAT NNN NNN NNa ttc att aag gtA GGA ATT GGC GGG GGA GCA C-3′, and 5′-AAT GAT ACG GCG ACC ACC GAG ATC TAC CAN NNN NNN Nta tgg taa ttc aGC GGT GTG TGC AAG GAG C-3′, made up of an Illumina adapter (underlined), an 8-nucleotide barcode indicated by NNNNNNNN, a pad region (10 nucleotides) and linker (2 nucleotides) shown in lower case, followed the archaeal 16S rRNA gene-specific regions (Casamayor et al., 2002; Skillman et al., 2004, respectively). Each sample was identified by the unique combination of the pair of 8-nucleotide Hamming barcodes (Hamady et al., 2008) in the forward and reverse primers. PCR products were sequenced using Illumina MiSeq 2 × 250 base PE V2 runs at the Massey Genome Service (Palmerston North, New Zealand). Quality control of the raw sequence data used BWA alignment at a 0.01 cutoff with the BWA trimming option in SolexaQA++ (Cox et al., 2010). Read 1 data were processed and analyzed using QIIME software v1.9.1 (Caporaso et al., 2010) with operational taxonomic unit (OTU) picking performed at 99% sequence identity. Representative sequences for each OTU were assigned taxonomic strings by BLAST (Altschul et al., 1990) using the blastn algorithm, with megablast option, and an e-value cutoff of 0.001 against an in-house database containing the SILVA V123 database (Quast et al., 2013; Henderson et al., 2019) which contains updated taxonomy for rumen bacteria, as well as archaeal 16S rRNA gene and protozoa 18S rRNA gene databases as previously described (Seedorf et al., 2015; Kittelmann et al., 2015). Sequences have been submitted to NCBI BioSample under accession numbers SAMN27064694 to SAMN27064740.

### Statistical analysis

The significance of differences between the groups was analyzed using One-way ANOVA followed by a Dunnett’s *post hoc* multiple comparison test (Stoline, 1981). All data are expressed as the mean ± SE and plotted using Minitab V.18.1 (Minitab Inc., United States). The level of significance was set at a *P*-value of ≤0.05.

## Results

### Preparation of immunomagnetic beads for capturing methanogens

Antibodies produced in sheep vaccinated with individual methanogen isolates (*M. ruminantium* M1, *Methanobrevibacter* sp. AbM4, *Methanobrevibacter* sp. D5, *Methanobrevibacter* sp. SM9), a mixture of all four isolates together (MIX4), or adjuvant alone, were used to coat paramagnetic beads. The concentrations of antibody bound to these paramagnetic beads and the surface antibody concentration on the paramagnetic beads are shown in Table 2. The results indicated that, for each type of antibody, greater than 90% antibody coupling was achieved.

**TABLE 2:** Antibody binding to IMB.

Antibodies^a^	Antibody applied (μg/mg bead)	Antibody bound^b^		Concentration of surface antibody (fg/μm^2^)^c^
		
		(μg/mg bead)	Percentage of applied	
SM9	40	37.9	94.8	10.2
D5	40	37.6	94.0	10.2
M1	40	37.5	93.8	10.1
AbM4	40	37.5	93.8	10.1
MIX4	40	38.2	95.5	10.3
NC	40	37.7	94.3	10.2

^a^Antisera were produced in sheep vaccinated with whole cells of M. ruminantium M1, Methanobrevibacter sp. AbM4, Methanobrevibacter sp. D5, Methanobrevibacter sp. SM9, a mixture of all four isolates (MIX4) or adjuvant alone (NC). The IgG fraction in antisera was enriched using HiTrap protein G HP columns (GE HealthCare) prior to use with IMBs.

^b^The amount of antibody bound to the IMBs was calculated from the differences between total applied prior to conjugation and that remaining after conjugation.

^c^1 mg of beads contain 1 × 10^9^ beads with a total surface area of 3.7 × 10^9^ μm^2^.

**FIGURE 1:**
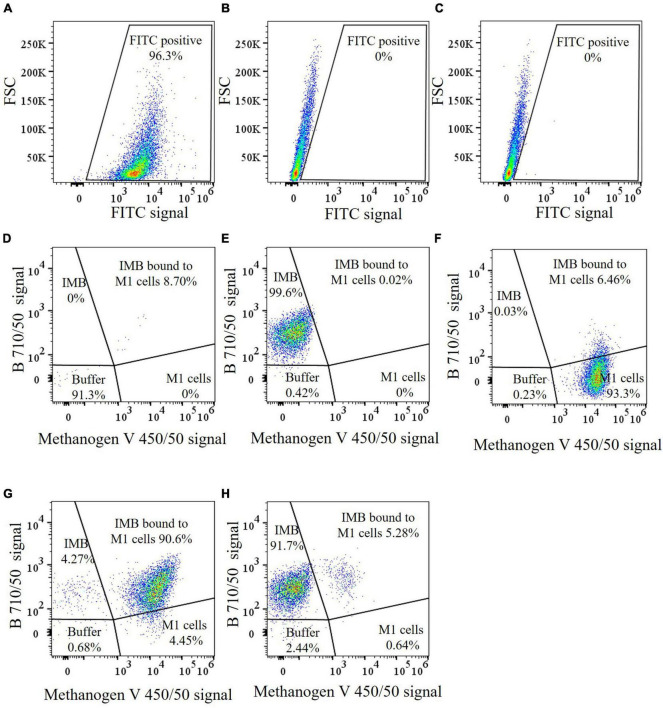
Flow cytometric analysis of IMBs preparations. **(A−C)** Coupling of anti-M1 serum antibodies to paramagnetic beads demonstrated using FITC labeled anti-sheep antibodies. A gate was selected for the FITC positive subset. **(A)** Dot plot of M1-IMBs coated with sheep antibodies were detected with secondary FITC-labeled anti-sheep antibodies, **(B)** Dot plot of forward scatter (FSC) vs FITC of M1-IMBs, **(C)** FITC-labeled anti-mouse antibodies were used as a negative control. **(D−H)** A violet laser was used to detect *M. ruminantium* M1 cells based on the auto fluorescence of methanogen coenzyme F_420_. IMBs were detected by the B710/50 detector. Gates were selected based on the position of individual sample components when analyzed separately. **(D)** Dot plot of B 710/50 vs methanogen V 450/50 of buffer only control, **(E)** IMBs only control, **(F)** *M. ruminantium* M1 cells, **(G)** M1-IMBs were incubated with M1 cells and analyzed after washing, M1 cells bound to M1-IMBs were clearly seen as a shift on the X-axis, **(H)** NC-IMBs were incubated with M1 cells and analyzed after washing. The sectors of the plots are labeled with the expected locations of different combinations of buffer particles, IMBs and methanogens, and the percentage values are the particle counts in that sector.

**FIGURE 2:**
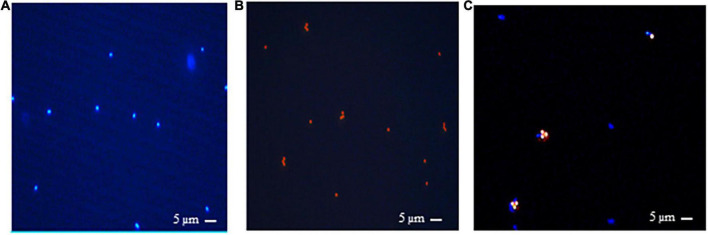
Fluorescence microscopic images showing rumen methanogens which naturally fluorescence under UV light due to presence of coenzyme F_420_ and emit a blue fluorescence with an excitation at 420 nm. **(A)** *M. ruminantium* strain M1, **(B)** 1-μm beads viewed under UV illumination, **(C)** *M. ruminantium* M1 binding to M1-IMBs viewed under UV light. Methanogens with blue fluorescence are visible in complexes with beads under UV illumination.

Antibodies produced against *M. ruminantium* M1 were used to optimize coupling of antibodies onto paramagnetic beads to produce IMBs. Paramagnetic beads were coated with IgG-enriched fractions from antisera produced against *M. ruminantium* M1 and used to assess binding of the cells to IMBs by flow cytometry. Ninety six percent of IMBs coated with sheep anti-M1 IgG (designated M1-IMBs) were detected following labeling with secondary FITC-labeled anti-sheep antibody (Figure 1A). M1-IMBs without anti-sheep secondary antibody or reacted with FITC-labeled anti-mouse antibody gave no signal (Figures 1B,C). Buffer alone and M1-IMBs displayed very low fluorescence at 450 nm (Figures 1D,E), but M1 cells alone produced strong signal at this wavelength due to their autofluorescence (Figure 1F). IMBs alone were detected due to their intrinsic autofluorescence when excited by 710 nm light (Figure 1E), while methanogens produced a lower signal at this wavelength (Figure 1F). The ability of M1-IMBs to bind to M1 cells was determined using both channels. When there was 10-fold excess of M1-IMBs, they bound more than 90% of M1 cells (Figure 1G). Beads coated with IgG from sheep vaccinated with adjuvant alone (NC-IMBs) bound fewer M1 cells (Figure 1H). In addition, fluorescence microscopy confirmed the binding of M1 cells to M1-IMBs (Figure 2).

### Capturing efficiency of immunomagnetic beads

**FIGURE 3:**
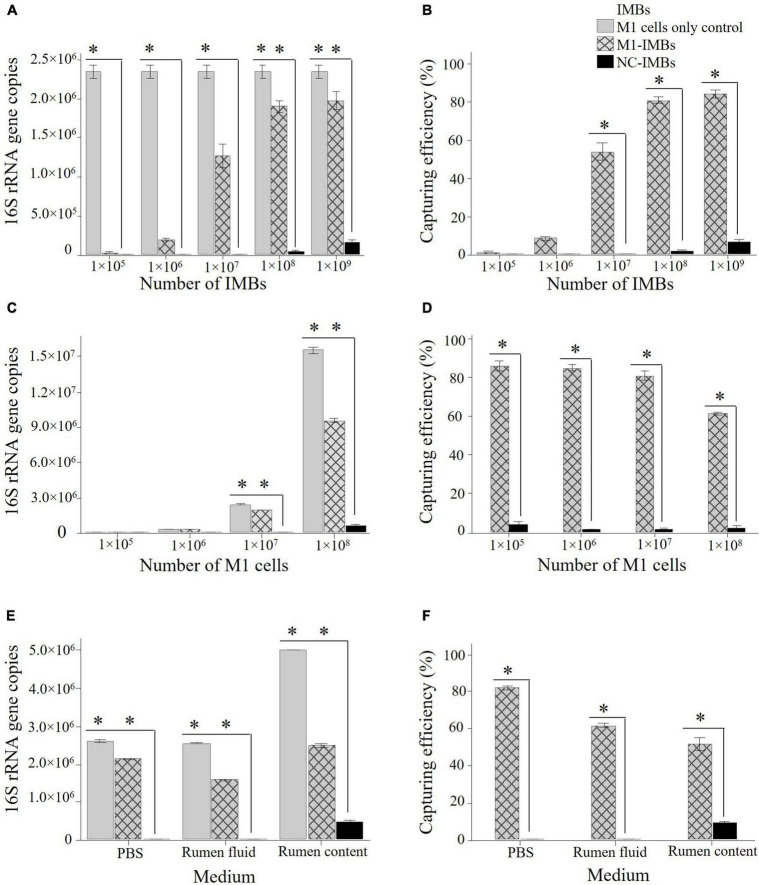
Optimization of IMBs to capture *M. ruminantium* M1 cells. Different numbers of M1-IMBs and M1 cells were used to optimize the number of IMBs required to capture maximum number of M1 cells. Genomic DNA was isolated from captured M1 cells and quantified by qPCR using primers to amplify 16S rRNA gene. Copies of 16S rRNA gene/μL of sample are plotted against each dilution of M1-IMBs or M1 cells. NC-IMBs were used as a control in this experiment. Capturing efficiency was calculated on the basis of initial number of M1 cells present in the sample and defined as (methanogen cell fraction bound calculated on the basis of 16S rRNA gene copies through qPCR)/(total number of methanogen cells present in the samples) × 100. Capturing efficiency of each sample is plotted against number of M1 cells used in the assay. **(A)** Different numbers of M1-IMBs were used to capture 1 × 10^7^ M1 cells. **(B)** Capturing efficiency (%) of different numbers of M1-IMBs were calculated to capture 1 × 10^7^ M1 cells. **(C)** 1 × 10^8^ M1-IMBs were used to capture different numbers of M1 cells. **(D)** Capturing efficiency (%) of 1 × 10^8^ M1-IMBs were calculated to capture different numbers of M1 cells. **(E)** 1 × 10^8^ M1-IMBs were used to capture 1 × 10^7^ M1 cells in PBS, rumen fluid or rumen content samples. **(F)** Comparison of the capturing efficiency (%) of 1 × 10^8^ M1-IMBs to capture 1 × 10^7^ M1 cells in PBS, rumen fluid or rumen contents. Data are presented as means (± SE) of three technical replicates. *significantly different to negative control (NC-IMBs) group (*P* < 0.05).

A range of serially diluted M1-IMBs from 1 × 10^9^ to 1 × 10^5^ was mixed with a fixed number of 1 × 10^7^ M1 cells (estimated based on OD_600_) to determine the optimum concentration of beads required to capture *M*. *ruminantium* M1 cells in PBS buffer. Captured M1 cells were quantified by qPCR using primers to amplify the archaeal 16S rRNA gene. Adding more than 1 × 10^8^ M1-IMBs did not markedly increase the binding efficiency toward 1 × 10^7^ M1 cells (Figures 3A,B), and so 1 × 10^8^ IMBs were used for all subsequent experiments. NC-IMBs (coated with the serum from control animals) captured significantly less M1 cells in comparison to M1-IMBs in parallel assays (Figures 3A,B). These results indicated high specificity and sensitivity of the M1-IMBs in capturing M1 cells.

To determine the maximum number of M1 cells bound or retained on the surface of M1-IMBs, 1 × 10^5^ to 1 × 10^9^ M1 cells were mixed with 1 × 10^8^ M1-IMBs in buffer (PBS). Eighty percent of 1 × 10^7^ M1 cells were captured by 1 × 10^8^ M1-IMBs (Figures 3C,D) as indicated by copies of 16S rRNA gene, compared to the starting cell suspension. This capturing efficiency decreased when more than 1 × 10^7^ M1 cells were added to the assay (Figures 3C,D). This indicated that almost 80% of M1 cells out of 1 × 10^7^ cells used at the start of the assay could be captured by the (1 × 10^8^) of M1-IMBs under these assay conditions.

The capturing efficiency of M1-IMBs was also assessed using freshly obtained sheep rumen fluid, from which microbes were first removed by filtration before 1 × 10^7^ M1 cells were added. A total of 1 × 10^8^ M1-IMBs captured 62% of these M1 cells from the rumen fluid (Figures 3E,F). A parallel control in buffer again gave capturing efficiency of 80%. This suggests that the unknown components of the rumen fluid somewhat impacted the capturing efficiency of M1-IMBs but did not abolish binding.

As expected, adding 1 × 10^7^ M1 cells to rumen content samples containing resident methanogens further reduced the capturing efficiency because as shown in Figure 3D, adding more methanogens (10^7^) had a negative impact on the capturing efficiency (Figures 3E,F).

### ICT can be used to determine cross-reactivity of antibodies produced against individual methanogen isolates

**FIGURE 4:**
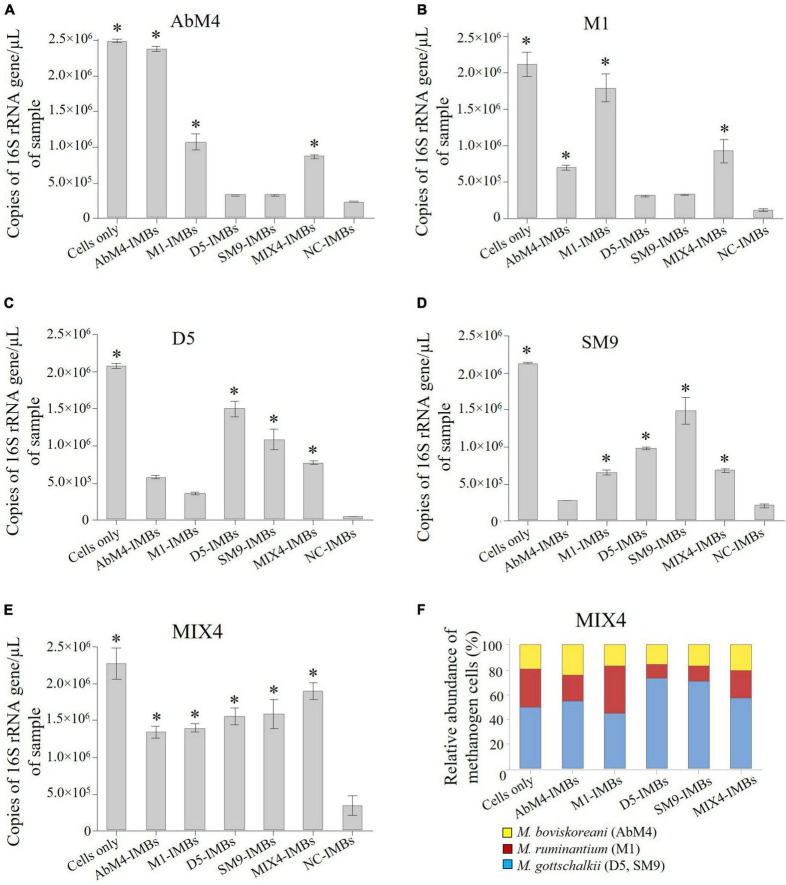
Using IMBs to determine cross-reactivity of antibody-coated beads in PBS buffer. 1 × 10^8^ IMBs that were coated with purified antisera produced in sheep against *M. ruminantium* M1, *Methanobrevibacter* sp. AbM4, *Methanobrevibacter* sp. D5, *Methanobrevibacter* sp. SM9, MIX4 and negative control (NC) sera were used to capture cells of each the *Methanobrevibacter* isolates separately. Genomic DNA was isolated from captured cells and quantified by qPCR using primers to amplify 16S rRNA gene. Copies of 16S rRNA gene/μL of sample are plotted against the types of IMBs used to capture the cells. NC-IMBs were used as a control, while samples containing only cells were used to determine the total count of cells present in the initial methanogen sample. **(A)** Capturing AbM4 cells using different IMBs; **(B)** Capturing M1 cells using different IMBs; **(C)** Capturing D5 cells using different IMBs; **(D)** Capturing SM9 cells using different IMBs; **(E)** Capturing MIX4 cells using different IMBs; **(F)** Relative abundance of methanogens captured by IMBs from a mix of the culture determined by the 16S rRNA gene sequencing, corrected for the number 16S rRNA gene copies present in the respective methanogen genomes. Data are presented as means (± SE) of three technical replicates. *significantly different to negative control (NC-IMBs) group (*P* < 0.05).

To evaluate cross-reactivity, IMBs were produced by coating the antibodies produced against the four methanogen isolates: *M. ruminantium* M1, *Methanobrevibacter* sp. AbM4, *Methanobrevibacter* sp. D5, and *Methanobrevibacter* sp. SM9. These IMBs were then tested for their ability to capture pure culture of each methanogen isolate, either individually or from a mixture of these four isolates. All four methanogen isolates were captured by their respective IMBs (Figures 4A–D). There was also comparable binding of the other *Methanobrevibacter* isolates to IMBs coated with antibodies raised against any of the isolates. Maximum capture was observed with the MIX4-IMBs which were coated with antibodies produced against a mix of all four *Methanobrevibacter* isolates (Figure 4E). Analysis of the cells captured by MIX4-IMBs from the mixture of the four isolates showed that these IMBs captured belonged to three broad species in the mix: *M. ruminantium* clade (isolate M1), *M. gottschalkii* clade (isolates D5 and SM9) and *M. boviskoreani* (isolate AbM4) (Figure 4F). Figure 4 illustrates the tendency of IMBs coated with antibodies against individual isolates to bind more cells from the species that the isolate belonged to.

### Capture of methanogens from sheep rumen content samples

**FIGURE 5:**
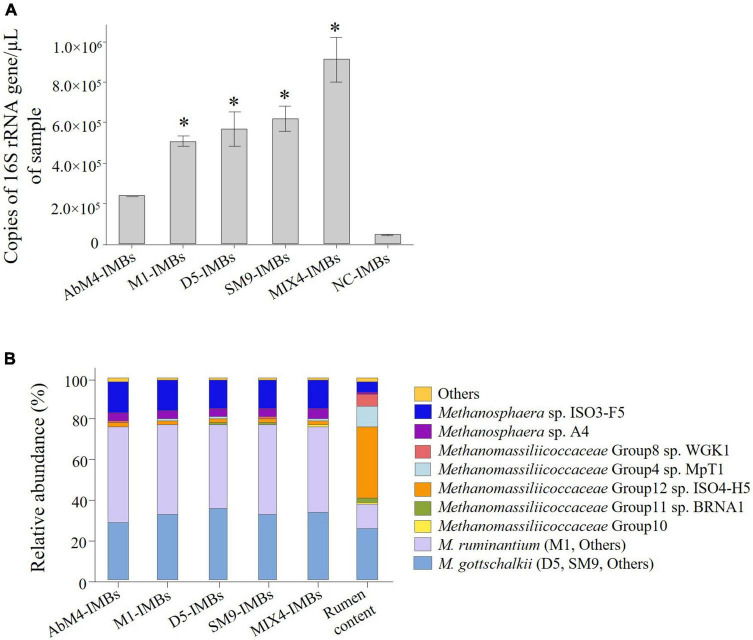
IMBs used to capture methanogens from rumen content samples. 1 × 10^8^ IMBs coated with antibodies produced against *M. ruminantium* M1, *Methanobrevibacter* sp. AbM4, *Methanobrevibacter* sp. D5, *Methanobrevibacter* sp. SM9, a mixture of all four methanogens (MIX4), or negative control (NC) sera were used to capture methanogen cells from rumen content samples. **(A)** Genomic DNA was isolated from captured cells and quantified by qPCR using primers to amplify 16S rRNA gene. Copies of 16S rRNA gene/μL of sample are plotted against types of IMBs used to capture the cells. NC-IMBs were used as a serum control, while rumen content only samples were used to get the total count of cells present in the initial sample. Data are presented as means (± SE) of three biological replicates. *Significantly different to negative control (NC-IMBs) group (*P* < 0.05). **(B)** Relative abundance of different methanogens captured by IMBs in rumen content samples.

To test the efficacy of this binding assay when performed in rumen contents, a total of 1 × 10^8^ IMBs were incubated with rumen content samples for 1 h at room temperature. The IMBs were washed, and DNA was then isolated from the captured methanogens for qPCR and DNA sequence analysis. The results showed that the IMBs coated with antibodies raised against the individual methanogen isolates and MIX-IMBs captured larger numbers of native methanogens from the rumen content samples compared to the NC-IMBs (Figure 5A). In addition, MIX4-IMBs captured the greatest number of rumen methanogens, suggesting that antibodies raised against the four methanogens could bind to different methanogens present in the rumen sample.

Analysis of 16S rRNA gene sequences of the captured cells showed that the IMBs captured not only members of the *M. ruminantium* clade, the *M. gottschalkii* clade, and *Methanobrevibacter boviskoreani* resident in the rumen content samples, corresponding to the isolates used to raise the antibodies coating the beads, but also members of the related genus *Methanosphaera* which is also classified with *Methanobrevibacter* in the family *Methanobacteriaceae* (Figure 5B). This suggests that antibodies raised against the four methanogens can cross-react to other methanogens species present in the rumen. IMBs also captured other minor species of *Methanobrevibacter* (*M. acididurans*, *M. arboriphilus*, *M. boviskoreani*, *M. smithii*, and *M. subterraneum*; totaling < 0.03%), and *Methanosphaera* (*M. cuniculi*, *Methanosphaera* Group5, and *M. stadtmanae*; totaling < 1%). Additionally, other low abundance methanogens captured were members of the family *Methanomassiliicoccaceae* (< 0.07%) and some other taxa (*Methanimicrococcus*, *Methanocorpusculum*, *Methanomicrobium*, and *Methanosaeta*; totaling < 0.002%).

## Discussion

In this study, we developed an immunomagnetic capture technology (ICT) assay for methanogens by coating paramagnetic beads with sheep polyclonal anti-methanogen antibodies and examining the ability of these to bind methanogens in buffer and rumen samples. ICT is easy to use and simple to implement in laboratories, with no need for highly specialized instrumentation. Recently, ICT has received considerable attention as an efficient and versatile tool to capture food borne pathogens from complex communities of microflora in food samples (Wang et al., 2011; Vinayaka et al., 2019). The orientation of immobilized antibodies on a surface is a critical factor in the binding efficiency between an antibody and its ligand, and appropriate orientation of antibodies maximizes their capacity for antigen recognition. Therefore, we used tosyl-activated magnetic beads in this study (Hermanson, 2013). The tosyl-activated magnetic beads provide reactive sulfonyl ester groups to covalently link antibodies via primary amino groups on their Fc region, which is the ideal orientation for antibodies in a manner that maximizes their ability to capture target analyte (Otieno et al., 2016). Using this variant of paramagnetic beads, a significant reduction in free antibody concentration was observed in the supernatant of the antibody-beads conjugate mixture after overnight incubation. This resulted in an antibody immobilization efficiency of more than 90%, which is in agreement with the manufacturer’s claim (Dynabeads^®^ MyOne™ Tosylactivated, Invitrogen) and a previous study (Tu et al., 2009).

The successful conjugation was further confirmed when IMBs-coated with specific anti-methanogen antibodies were found to bind the respective target methanogen isolates to which the antibodies were raised and exhibited capture efficiencies of more than 80% in buffer when the IMBs were incubated in suspensions of pure methanogen cells. These results indicated the anti-methanogen antibodies bind to surface exposed antigens of methanogens. Non-specific or background capture using antibodies from control sheep or non-coated beads was less than 5% of the capture using IMBs coated with specific antibodies illustrating the specificity of this technique.

We observed a lower binding efficiency (roughly 60%) in rumen fluid. ICT capturing efficiency was reduced a further 10% in rumen contents. The lower recovery of methanogens from rumen fluid and rumen contents probably resulted at least in part from the higher viscosity of rumen fluid relative to buffer, and this may have disturbed the magnetic separation and led to losses of captured magnetic beads. Diluting the rumen fluid or changing the sampling time before ICT separation may help to improve capture efficiency by reducing viscosity and stringency. In addition, eliminating the complex matrices from rumen content samples by differential centrifugation may help to improve capturing efficiency of microbes but will reduce the capture of microbes bound to feed particles (Rodriguez et al., 2003; Valle et al., 2015) that get lost during this procedure.

A degree of cross-reactivity was observed when IMBs were incubated with cells from isolates that were different from the one used to produce the IMBs. This cross-reactivity indicates the presence of antigens that are common to species of *Methanobrevibacter*. For example, antigenic similarities between the pairs *M. ruminantium* M1 and *Methanobrevibacter* sp. AbM4, as well as *Methanobrevibacter* sp. D5 and *Methanobrevibacter* sp. SM9 were noticeable with the capturing ability of their respective IMBs in the mixed cell preparation and in rumen content samples. Antigenic similarities between SM9 and D5 are likely to be explained by the fact that both belong to the *M. gottschalkii* clade of *Methanobrevibacter*. Similarly, 16S rRNA analysis suggests that the *M. boviskoreani* clade, to which AbM4 belongs, is closely related to the *M. ruminantium* clade (which includes M1) than to the *M. gottschalkii* clade (Seedorf et al., 2015). IMBs also captured other species of the genus *Methanobrevibacter* and members of the related genus *Methanosphaera*, which also belongs to the family *Methanobacteriaceae* within the order *Methanobacteriales*. This indicates the presence of family-wide common antigens. In contrast, IMBs coated with antibodies raised against *Methanobrevibacter* spp. co-captured few members of the family *Methanomassiliicoccaceae* and of other methanogens not in the family *Methanobacteriaceae* in the rumen content samples, even though these made up about half the total population of the rumen methanogens. This indicates that surface-exposed antigens are not shared between these very distantly related families of rumen methanogens and demonstrates that it is possible to target specific subsets of methanogens.

Vaccinology usually aims to harness antibodies that target single species, but some vaccines provide hosts with simultaneous protection against different members of a genus, e.g., clostridial vaccines (Alam et al., 2008). To develop a broad-spectrum anti-methanogen vaccine to mitigate livestock methane emission, it would be useful to understand cross-reactivity of antibodies generated against selected antigens. ICT promises to be a useful tool in this regard. The combined approach used in this study, to capture methanogens from rumen samples using antibodies immobilized on paramagnetic beads, and subsequent qPCR and sequencing for their quantification and identification, provides a rapid, and relatively simple method to determine the specificity or cross-reactivity of methanogen antigens and their distribution in cultured and uncultured rumen methanogens.

## Data availability statement

The datasets presented in this study can be found in online repositories. The names of the repository/repositories and accession number(s) can be found below: https://www.ncbi.nlm.nih.gov/, PRJNA821247.

## Ethics statement

The animal study was reviewed and approved by Grasslands Animal Ethics Committee (Palmerston North, New Zealand).

## Author contributions

SK conducted the major ICT experiments and qPCR analysis. SG and TW conducted animal trial and performed sera and rumen content collection. JMR contributed to design of flow cytometry experiments. SB performed DNA isolation and qPCR. SK and PJ performed fluorescence microscopy. RH prepared the methanogen cell cultures. MRK processed the sample for DNA sequencing. PJ processed and analyzed the sequence reads. SK wrote the original draft. AH, DW, PJ, SG, and SK reviewed and edited the manuscript. DW, PJ, and AH conceived and supervised the study. All authors have read and agreed to the published version of the manuscript.
